# Polarimetric image recovery method combining histogram stretching for underwater imaging

**DOI:** 10.1038/s41598-018-30566-8

**Published:** 2018-08-20

**Authors:** Xiaobo Li, Haofeng Hu, Lin Zhao, Hui Wang, Yin Yu, Lan Wu, Tiegen Liu

**Affiliations:** 1 School of Precision Instrument & Opto-electronics Engineering, Tianjin University, Tianjin, 300072 China; 2 Institute of Optical Fiber Sensing of Tianjin University, Tianjin, 300072 China; 3 Tianjin Optical Fiber Sensing Engineering Center, Tianjin, 300072 China; 4 Key Laboratory of Opto-electronics Information Technology, Ministry of Education, Tianjin, 300072 China; 5 State Key Laboratory of Modern Optical Instrumentation, Zhejiang University, Zhejiang, 310027 China

## Abstract

The underwater imaging could be severely degraded by the scattering media because of the backscattered light and signal attenuation, especially in the case of strong scattering for dense turbid medium. In this paper, we propose an improved method for recovering the underwater image combining the histogram stretching and polarimetric recovery in a proper way. In this method, we stretch the histograms of the orthogonal polarization images while maintaining the polarization relation between them, and then, based on the processed orthogonal polarization images, the recovered image with higher quality can be obtained by the traditional polarimetric recovery method. Several groups of experimental results demonstrate that the quality of underwater images can be effectively enhanced by our method, and its performance is better than that of the traditional polarimetric recovery method. In particular, the proposed method is also quite effective in the condition of dense turbid medium.

## Introduction

Due to the scattering and absorption by the particles existing in water, the underwater image could be blurred and the image quality is reduced^1–8^. It is subsequently a main problem in various underwater applications. For example, inspecting the ship hulls and exploring the marine resources^9,10^. Therefore, the effective way for enhancing the quality of underwater images is significant for these demands.

Various methods have been developed for the quality enhancement of underwater images, which can be classified into two categories, one is based on the computer vision^11–16^. For example, histogram stretching^11^ is a quite simple and effective method for image enhancement. However, the drawback of this method is undifferentiating between different pixels, which could result in the blurring of the interested details^11^. He *et al*. proposed the dark channel prior (DCP) method, which initializes the transmission map and followed by refinement through soft matting^14^. Although DCP method yields the improved performance for image dehazing, the high computational complexity makes the entire process relatively time-consuming^15^. Kim *et al*. proposed an optimized contrast enhancement with a novel estimation of the transmittance by introducing a cost function, it can effectively remove haze and is also available for video dehazing^16^, but it may lead to the loss of details and edges in the dehazed image^12^. The other category of image recovery is based on the polarimetric model. The traditional polarimetric recovery method proposed by Schechner *et al*.^17,18^ is found to be an effective way to enhance the quality of hazy images in turbid medium, which recovers the image based on two images captured at orthogonal polarization states. Based on Schechner’s method, various improved methods have been proposed to recovery the hazy images^19–26^. For example, Liang *et al*. proposed an effective polarimetric dehazing method^21,24^ based on the angle of polarization (AOP), but this method needs four images captured at four different polarization states, which would increase the complexity of the system. In addition, the methods mentioned above mainly focus on the turbid medium that is not dense enough. In the case of dense turbid medium, the useful object signals are severely attenuated and a large amounts of backscattered lights are fused into detector, which makes it more difficult to get a satisfactory performance by these methods. Therefore, it is important to find a simple and effective recovery method for underwater imaging. To our knowledge, the combination of computer vision and polarimetric recovery method has not been considered yet, and we believe a proper combination of them could enhance the image quality more effectively, especially for the dense turbid medium.

In fact, the range of gray level of the image is compressed into a narrow band in turbid medium, especially in the condition of dense turbid medium. In this case, we propose an improved method for recovering the underwater image based on a proper combination of histogram stretching and polarimetric recovery. By the histogram stretching and polarization relating, the visibility of the two orthogonal polarization images are improved, and then the recovered image can be obtained by performing the traditional polarimetric method based on the processed orthogonal polarization images. In addition, several groups of experiments for different scenes and with different densities of turbid medium are performed, especially for the dense turbid medium, which verify the superiority and effectiveness of our method.

## Methodology

### Underwater polarimetric imaging model

**Figure 1:**
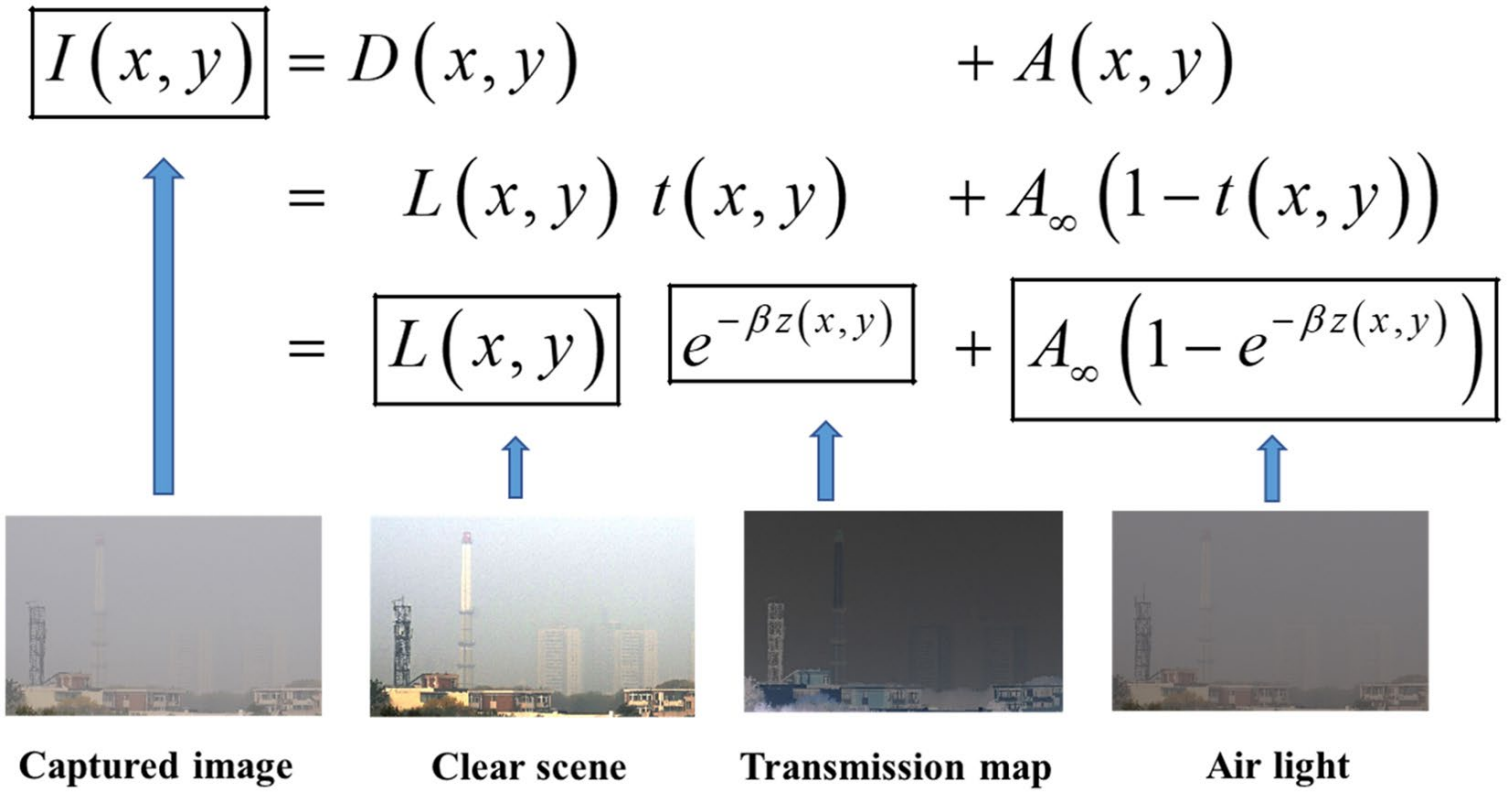
Image formation and visual example of illumination components through the turbid medium.

The typical model of imaging in turbid medium^17,18,27^ (such as fog, haze, turbid water, etc.) is illustrated in Fig. 1.

As shown in Fig. 1, the irradiance received by the detector *I*(*x, y*) (where (*x, y*) denotes the pixel coordinate) is the superposition of two components: (1). *Direct transmission* $D(x,y)=L(x,y)t(x,y)$, which represents the effect of scattering of light and the eventual decay of light before it reaches the detector, and (2). *Backscattered light* $A(x,y)={A}_{\infty }(1-t(x,y))$, which denotes the undesired lights received by the detector due to the scattering of particles. The term ${A}_{\infty }$ refers to the value of backscattered light from infinity in turbid medium, which is assumed to be a global constant being independent from location (*x, y*). The term *L*(*x, y*) denotes the haze-free radiance, which is not being scattered and attenuated by the scattering medium. Besides, the term *β* is the attenuation coefficient. In our case, we assume that *β* is spatially invariant. Therefore, the medium transmittance *t*(*x, y*) is determined by the propagation distance *z*(*x, y*) between the object and the detector^17^.

According to the polarimetric recovery method proposed by Schechner, we employ a linear polarizer before the light source to generate the linearly polarized illumination, and a rotating linear polarizer (analyzer) in front of detector. By rotating the analyzer to the parallel and orthogonal polarization states with the polarized illumination, one can get the co-linear image

1 $${I}^{\parallel }(x,y)=\frac{D(x,y)}{2}+{A}^{\parallel }(x,y),$$

and the cross-linear image

2 $$\,{I}^{\perp }(x,y)=\frac{D(x,y)}{2}+{A}^{\perp }(x,y),$$

respectively. The degree of polarization (DOP) of the backscattered light *A* is given by ${p}_{A}=({A}^{\parallel }-{A}^{\perp })/({A}^{\parallel }+{A}^{\perp })$^17^. The backscattered light for each pixel is estimated by:

3 $$A(x,y)=\frac{{I}^{\parallel }(x,y)-{I}^{\perp }(x,y)}{\,{p}_{A}}{\rm{.}}$$

Consequently, the transmission can be estimated by:

4 $$t(x,y)=1-\frac{A(x,y)}{{A}_{\infty }},$$

and finally, all these parameters are involved in the polarimetric model to obtain the recovered image as:

5 $$L(x,y)=\frac{I(x,y)-A(x,y)}{1-A(x,y)/{A}_{\infty }}=\frac{{I}^{\parallel }(x,y)+{I}^{\perp }(x,y)-A(x,y)}{1-A(x,y)/{A}_{\infty }}.$$

According to Eqs (3) and (5), only $\,{I}^{\parallel }$, ${I}^{\perp }$, ${A}_{\infty }$ and *p*_*A*_ are required to obtain the recovered image. The polarimetric recovery method is based on two orthogonal polarization images. However, previous polarimetric recovery methods directly process the original orthogonal polarization images^17,25^. Actually, although the polarizer can partially block the backscattering light, some unwanted scattering lights still exist in the orthogonal images, which results in the compression of gray level range of the intensity image^11^. Therefore, the quality of images can be enhanced if the range of gray level can be stretched.

### Recovery method combining histogram stretching

**Figure 2:**
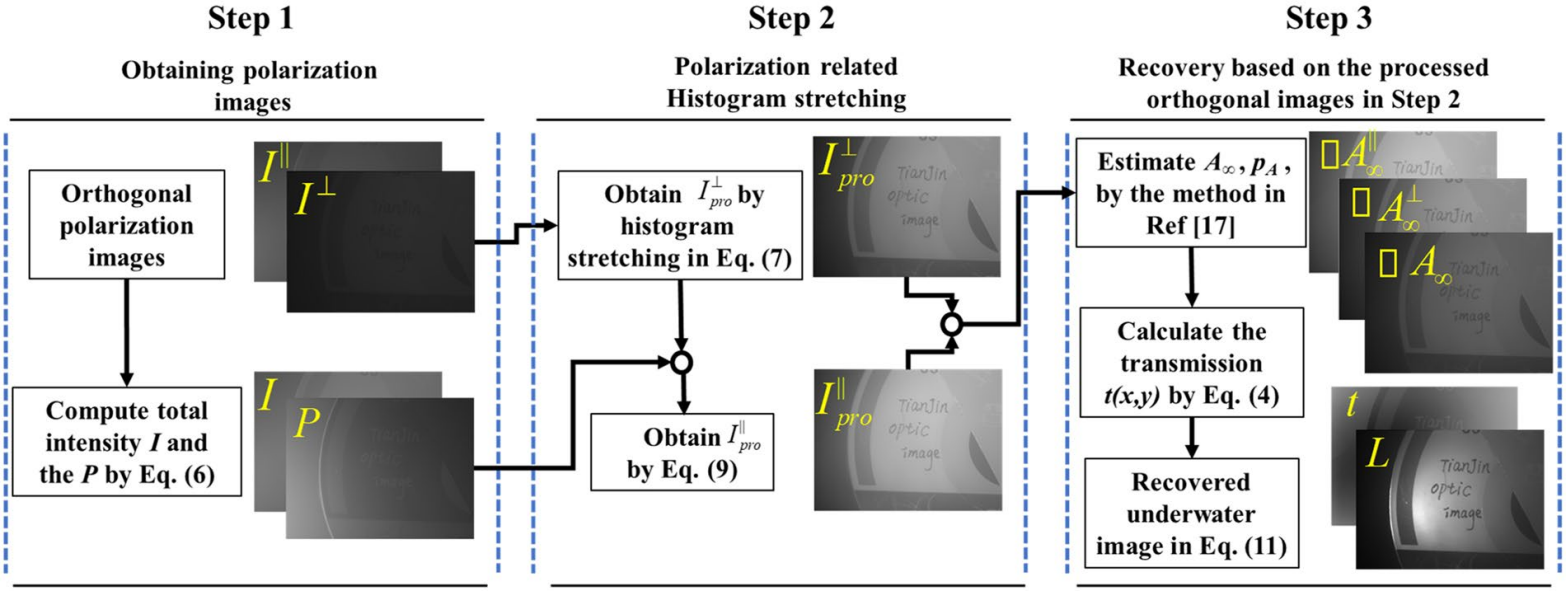
Flowchart of the proposed method combining histogram stretching and polarimetric recovery.

Combining the traditional polarimetric recovery method mentioned above and the histogram stretching method^11^, we propose an improved polarimetric method for underwater image recovery. The scheme for the proposed method is described in Fig. 2, which contains three steps:

### Step 1: Obtaining polarization images

In the first step, by adjusting the linear polarizer before CCD to the parallel and orthogonal polarization states with the polarized illumination, we obtain the co-linear image ${I}^{\parallel }(x,y)$ and the cross-linear image $\,{I}^{\perp }(x,y)$, and then the total intensity image and the DOP image of the acquired irradiance are calculated by:

6 $$I(x,y)={I}^{\parallel }(x,y)+{I}^{\perp }(x,y),\,P(x,y)=\frac{{I}^{\parallel }(x,y)-{I}^{\perp }(x,y)}{{I}^{\parallel }(x,y)+{I}^{\perp }(x,y)}.$$

### Step 2: Polarization related histogram stretching

The second step aims to stretch the histograms of the orthogonal polarization images. For the imaging in turbid medium, the range of gray level of image is compressed into a narrow band. The compression of the range of gray level can be alleviated by performing the histogram stretching method^11^. It needs to be clarified that there exists the polarization relation between the two orthogonal polarization images, and thus stretching the ranges of gray level of the two orthogonal polarization images independently could break this polarization relation. Indeed, DOP is the most important and basic polarimetric parameter, which is frequently used to describe the polarization property, and therefore, we consider DOP as the polarization relation of the two orthogonal polarization images. In order to keep the polarization relation, we need to process only one of the orthogonal images by performing histogram stretching method firstly, and then process the other one by the relevance of DOP. In this paper, we firstly process the cross-linear image ${I}^{\perp }(x,y)$ by performing histogram stretching as^11^:

7 $${I}_{pro}^{\perp }(x,y)=\frac{{I}^{\perp }(x,y)-min({I}^{\perp }(x,y))}{max({I}^{\perp }(x,y))-min({I}^{\perp }(x,y))}.$$

According to the Eq. (6), the polarization relation between the two orthogonal polarization images can be expressed based on the DOP as:

8 $${I}^{\parallel }(x,y)=\frac{1+P(x,y)}{1-P(x,y)}{I}^{\perp }(x,y),$$

and then the processed co-linear image is given according to Eqs (7) and (8) by:

9 $${I}_{pro}^{\parallel }(x,y)=\frac{1+P(x,y)}{1-P(x,y)}{I}_{pro}^{\perp }(x,y).$$

It needs to be clarified that the DOP of the two processed orthogonal polarization images by our method is kept the same as that of the two original orthogonal polarization images, while if we directly stretch the histograms of the two orthogonal polarization images independently, this polarization property could not be maintained. Besides, we can also firstly process the co-linear image instead of the cross-linear image by performing histogram stretching, and then processing the cross-linear image by the relevance of DOP. However, the cross-linear image is captured when the polarizer blocks more backscattered light, which means it is less influenced by the backscattered light than the co-linear image^5^, and thus the useful information is better kept in the cross-linear image. Therefore, it would be more effective to perform histogram stretching for the cross-linear image than that for the co-linear image.

### Step 3: Recovery based on the processed orthogonal images

Finally, in the third step we employ the processed images ${I}_{pro}^{\parallel }(x,y)$ and $\,{I}_{pro}^{\perp }(x,y)$ to estimate *p*_*A*_ and ${A}_{\infty }$ based on the traditional polarimetric recovery method^17^. As illustrated in Section 2, the recovered image of $L(x,y)$ can be then obtained by Eq. (5).

However, it can be seen that the denominator in Eq. (5) might be close to zero if *A* for some pixels are approximately close to $\,{A}_{\infty }$, which would lead to huge error to $L(x,y)$, and result in the “overamplification” in a local area of the recovered image^17,24^. The “overamplification” of distant objects can be reduced if we modify ${A}_{\infty }$ by multiplying it by a factor *ε* slightly greater than one as:

10 $${\hat{A}}_{\infty }=\varepsilon {A}_{\infty }.$$

Then one gets the recovered image as:

11 $$L(x,y)=\frac{I(x,y)-A(x,y)}{1-A(x,y)/\varepsilon {A}_{\infty }}.$$

In our work, it is found that by setting the value range of *ε* within [1, 1.2], one can get a good performance of image recovery.

## Experimental Results

**Figure 3:**
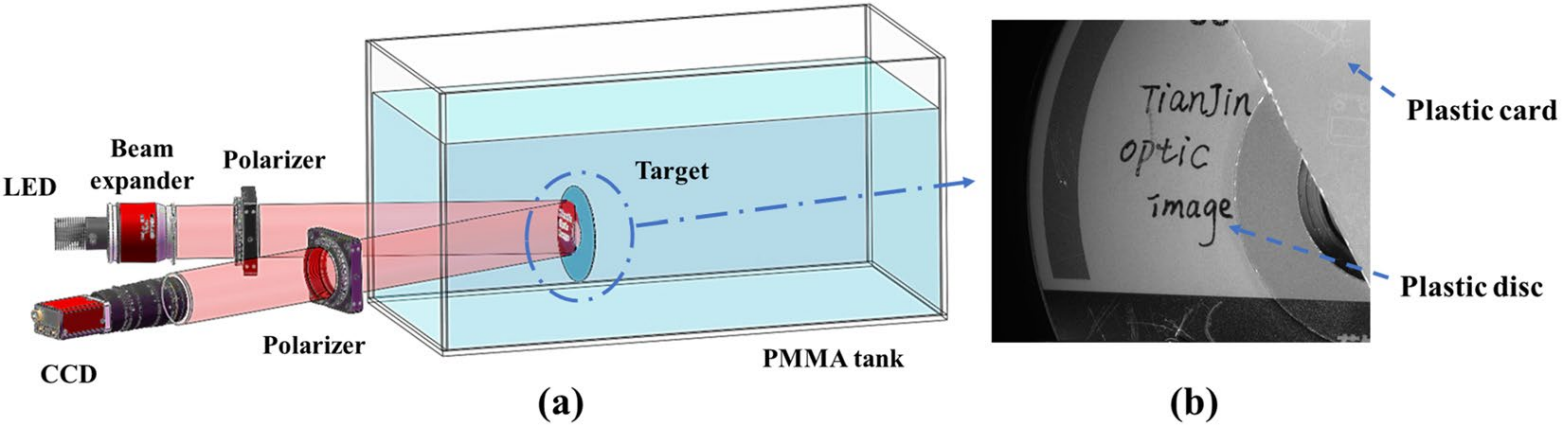
(**a**) Experimental setup; (**b**) Intensity image in clear water.

In the real-world experiments, we focus on the condition of underwater imaging in turbid medium. Figure 3(a) shows the experimental setup. We employ Light-Emitting Diode (LED) together with an optical filter to generate the active illumination light with the central wavelength of 632.8 nm. We employ a linear polarizer before LED light source to generate the linearly polarized illumination. Besides, and a rotating linear polarizer (analyzer) is placed in front of CCD.

We employ a transparent polymethyl methacrylate (PMMA) tank filled with water, and the milk (with protein content 3.6 g/100 ml, fat content 4.4 g/100 ml) is added in the water (with the volume of 60 cm × 25 cm × 20 cm) to generate the turbid medium. By adding different volumes of milk, one can generate the relatively slight and dense turbid media respectively. In order to verify the capability of our method, we perform several experiments for different scenes and with different densities of turbid medium, especially in dense turbid medium.

### Relatively slight turbid medium

**Figure 4:**
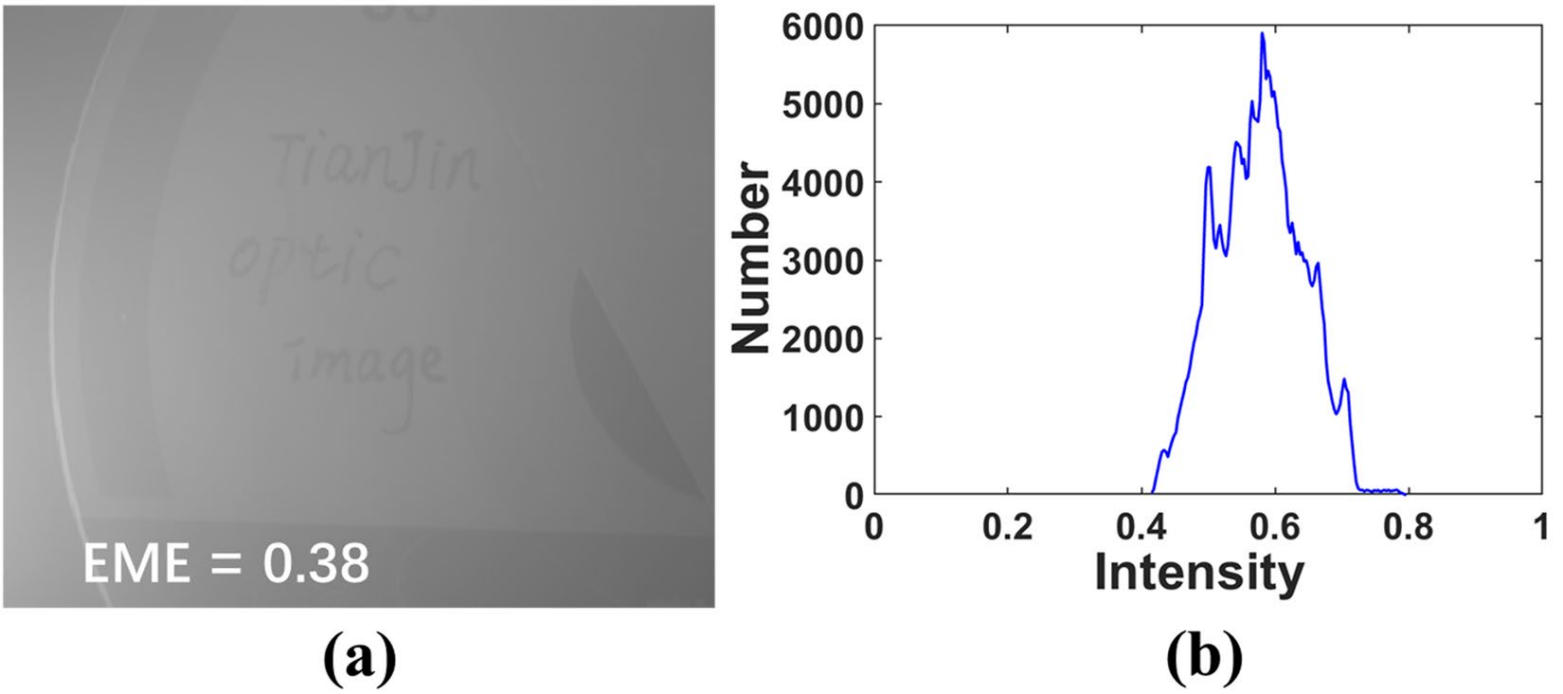
(**a**) The original underwater intensity image and (**b**) the histogram of intensity.

We add about 15 ml milk into the water to generate the relatively slight turbid medium. The target region consists of a plastic card stuck to a plastic disc, and Fig. 3(b) shows the clear image of this scene. When we add 15 ml milk in the water, the original underwater intensity image captured by CCD is presented in Fig. 4(a). In addition, the histogram of the intensity image is also presented in Fig. 4(b).

Comparing with the image in clear water in Fig. 3(b), the visibility of the image in Fig. 4(a) is poorer, and the details are severely degraded. In addition, according to the histogram in Fig. 4(b), the range of gray level of the intensity image is compressed into a narrow band. Here we introduce the measure of enhancement (EME) to quantify the image quality^25,26^ as follows:

12 $$EME=|\frac{1}{mn}\sum _{l=1}^{n}\,\sum _{k=1}^{m}\,20\,\mathrm{log}\,\frac{{i}_{{\rm{\max }};k,l}^{\omega }(x,y)}{{i}_{{\rm{\min }};k,l}^{\omega }(x,y)+q}|,$$

where the image *i*(*x, y*) is divided into *m* × *n* blocks $\,{i}_{k,l}^{\omega }$, ${i}_{{\rm{\max }};k,l}^{\omega }$ and ${i}_{{\rm{\min }};k,l}^{\omega }$ refer to the max- and min-value of the image in the block $\,{i}_{k,l}^{\omega }$. In order to avoid being divided by zero, the parameter *q* is a small constant set to 0.0001^25^. A high value of EME could indicate a high quality of the image. The value of EME of the original intensity image in Fig. 4(a) is calculated to be 0.38. This low value of EME also means a poor quality of the image in Fig. 4(a).

**Figure 5:**
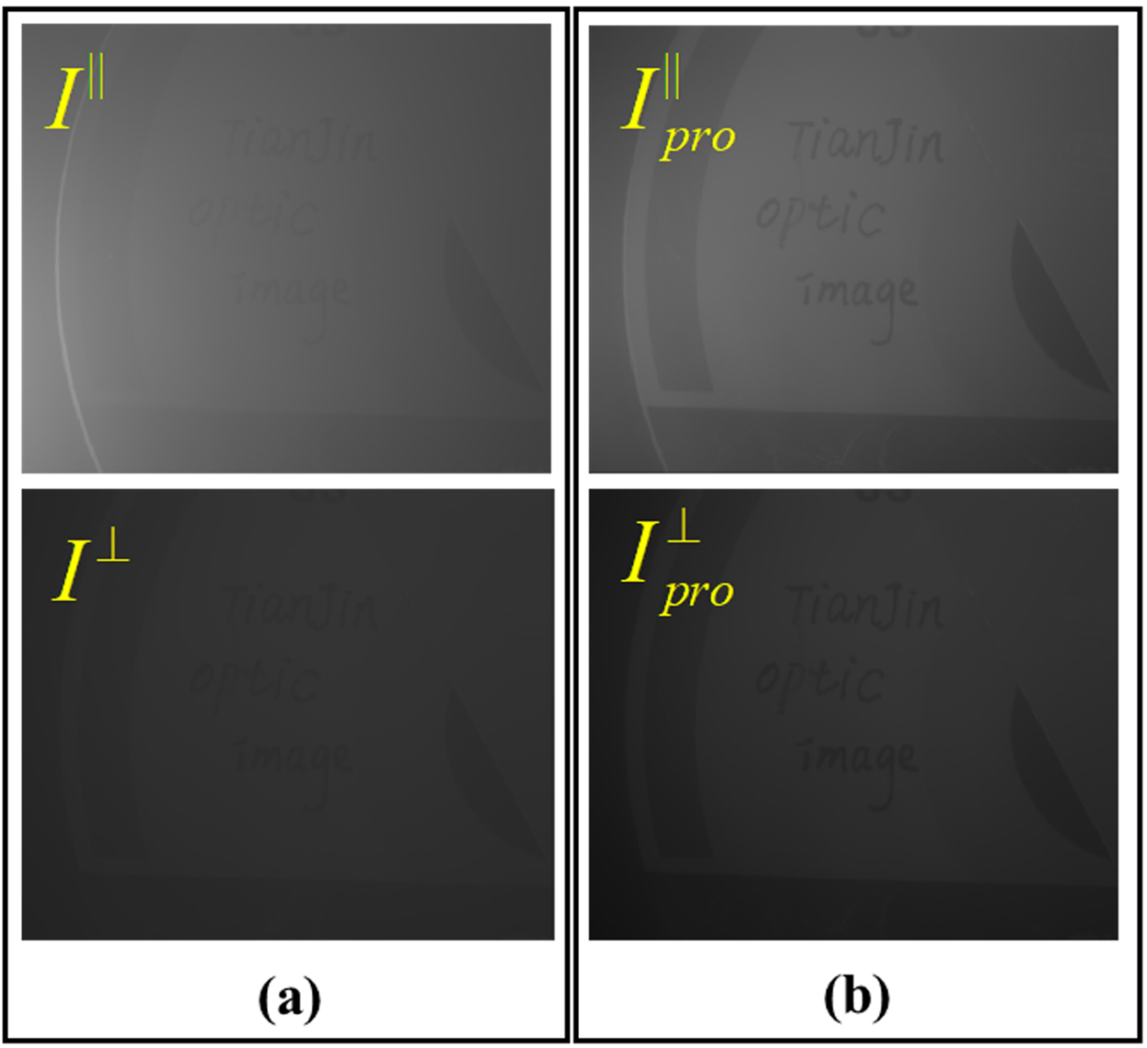
(**a**) Orthogonal polarization images; (**b**) Processed polarization images.

By adjusting the orientations of the linear polarizer in front of CCD, we obtain the two orthogonal polarization images ${I}^{\parallel }(x,y)$ and $\,{I}^{\perp }(x,y)$, which are presented in Fig. 5(a). According to the proposed method, we perform the histogram stretching for the cross-linear image ${I}^{\perp }(x,y)$ firstly, and according to Eqs (7) and (9), one can obtain the two processed orthogonal polarization images ${I}_{pro}^{\parallel }(x,y)$ and ${I}_{pro}^{\perp }(x,y)$, which are presented in Fig. 5(b).

Comparing Fig. 5(a,b), it is interesting to see that the processed orthogonal polarization images by performing histogram stretching and polarization relating are more clearly than the original ones. In addition, the detail information becomes more distinguishable, which is favorable for the following recovery process. According to Step 3, ${I}_{pro}^{\parallel }(x,y)$ and $\,{I}_{pro}^{\perp }(x,y)$ are employed to recovery the image by the traditional polarimetric recovery method.

**Figure 6:**
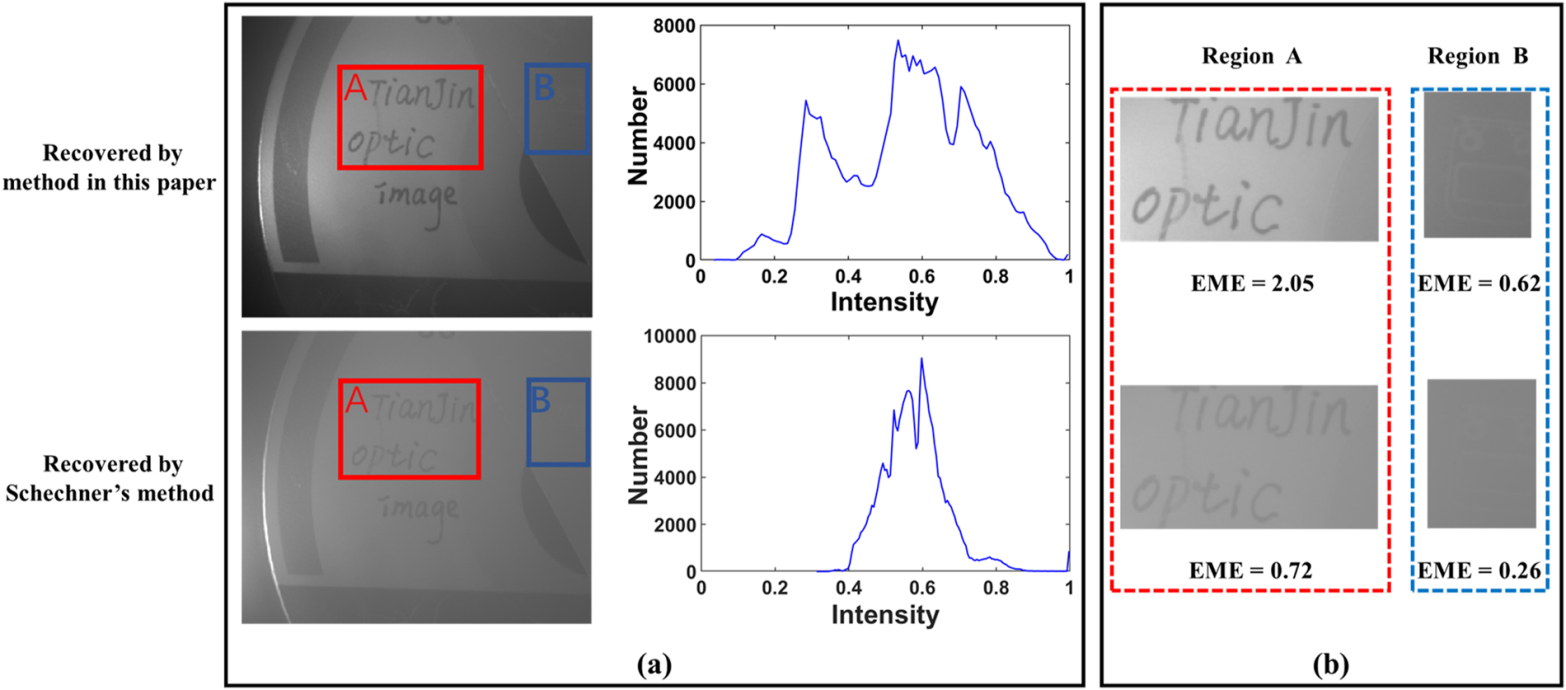
Recovered images by the proposed method in this paper and Schechner’s method in ref.^17^. The corresponding histograms of the intensity are presented in (**a**). The enlarged views of the red and blue rectangles are shown in (**b**).

Figure 6 shows the recovered image of the target object by employing the proposed method (with the value of the heuristic factor *ε* equal to 1.15). The recovered image by Schechner’s method is also presented in Fig. 6(a) for comparison. In addition, the histograms of the recovered images are also presented in the right of Fig. 6(a). It shows that the visibility of image is improved, and the range of gray level of the recovered image for the proposed method is wider than that for Schechner’s method, which indicates that the quality of image is enhanced. Besides, it is calculated that the EME value of the proposed method (EME = 2.50) is higher than that of Schechner’s (EME = 1.06), which also indicates a better image quality for our method.

Besides, the enlarged views of two details (A, B in red and blue regions, respectively) of the images recovered by our method and Schechner’s method are also shown in Fig. 6(b). It can be seen in Fig. 6(b) that the performances of different regions of the proposed method are better than that of Schechner’s method. In particular, the visibility of the weak detail (such as the detail in Region B) is also enhanced. Besides, the EME values of the two regions are also shown in Fig. 6(b), and it can be seen that the EME values of our method are higher than that of Schechner’s. All the experimental results in Fig. 6 demonstrate the effectiveness of our method in the condition of relatively slight turbid medium.

### Dense turbid medium

**Figure 7:**
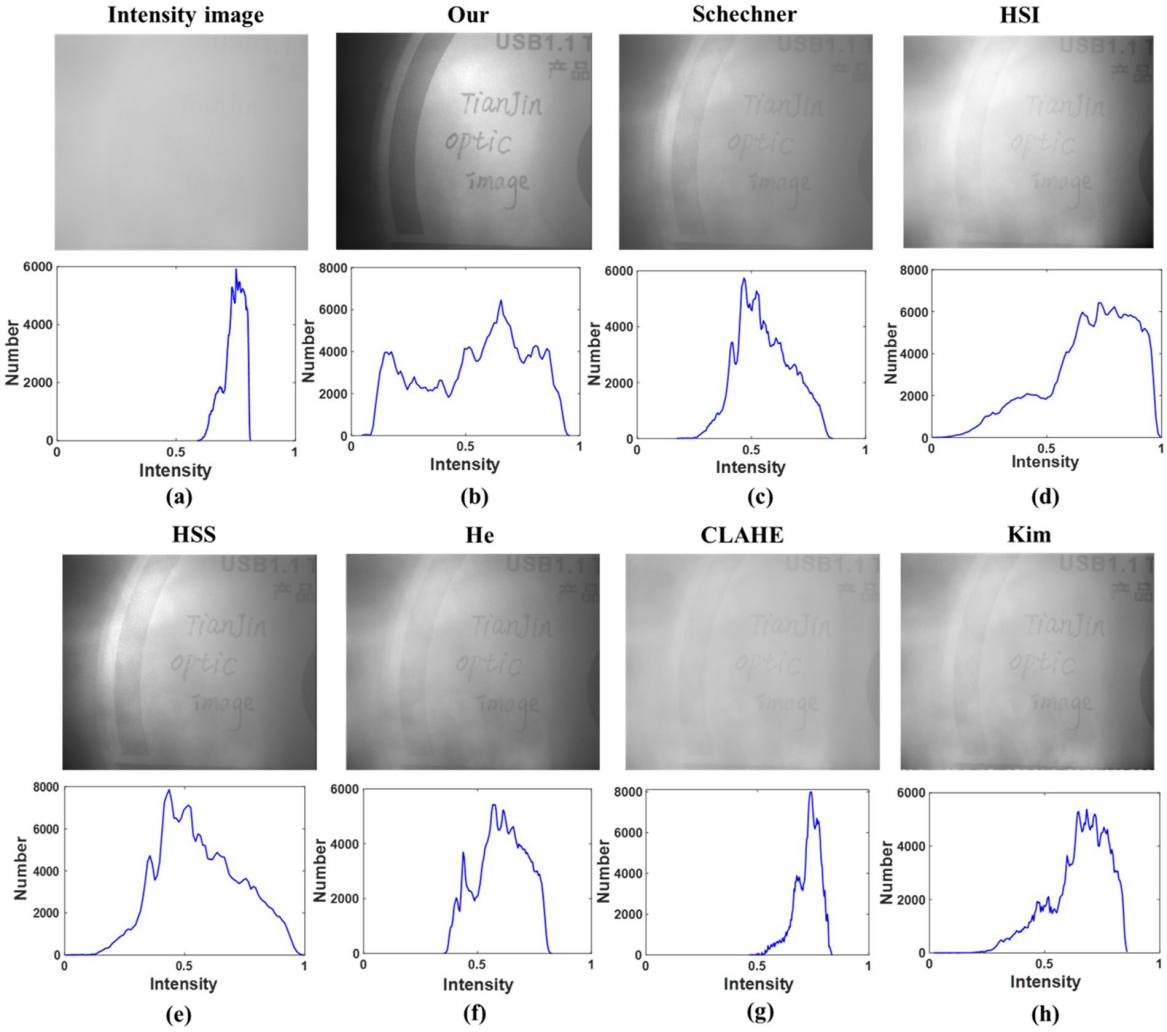
(**a**) Original intensity image; Recovered image by (**b**) our method; (**c**) Schechner’s method in ref.^17^, (**d**) performing histogram stretching directly for original intensity image; (**e**) performing histogram stretching for Schechner’s recovered image; (**f**) He’s method in ref.^14^, (**g**) CLAHE in ref.^13^, (**h**) Kim’s method in ref.^16^. The histograms of intensity are also presented in the blow row.

To verify the effectiveness of the proposed method in the condition of dense turbid medium, we add about 25 ml milk into the water (with the volume of 60 cm × 25 cm × 20 cm) to generate the dense turbid medium. The target is a plastic disc and the intensity image in turbid medium is presented in Fig. 7(a). Comparing with the “dense haze” cases in the previous works^7,24^, the visibility of the scene in Fig. 7(a) is even poorer, and the details are almost invisible. Therefore, we consider the turbid medium in Fig. 7 is dense. The recovered images by our method (with the value of the heuristic factor *ε* equal to 1.2) and Schechner’s method are shown in Fig. 7(b) and Fig. 7(c) for comparison. Besides, in order to show the effect of histogram stretching, we also present in Fig. 7(d) and Fig. 7(e) the result of histogram stretching for original intensity image (HSI) and the result of histogram stretching after Schechner’s method (HSS) respectively.

In addition, the results of some other representative methods for dehazing, including He’s dark channel prior^14^, contrast limited adaptive histogram equalization (CLAHE)^13^ and Kim’s contrast enhancement^16^, are also considered for comparison, which are presented in Fig. 7(f–h). These three methods are not developed based on the polarimetric model. Comparing the images in Fig. 7(a–h), it can be seen that the visibility of the recovered image by the proposed method presented in Fig. 7(b) is obviously better. In particular, comparing with the performance of Schechner’s method in Fig. 7(c), the performance of our method in Fig. 7(b) has a significant improvement, while that of HSS method in Fig. 7(e) has only a slight improvement. This is because our method perform histogram stretching for the cross-linear image, which contains more useful information and has a larger space of enhancement by histogram stretching^5,25^. Therefore, the proper way of combining histogram stretching and polarimetric image recovery is critical for the performance of image recovery, which is the key point of our method.

Besides, the histograms of these images are also presented in Fig. 7. Comparing all the histograms in Fig. 7, it can be seen that the histogram of our recovered image in Fig. 7(b) is closer to a relatively flat uniform histogram than others, and it almost covers the entire gray range, which could indicate a better image quality^28,29^. These experimental results in Fig. 7 demonstrate that our polarimetric recovery method is quite effective in dense turbid medium.

In addition, to further verify the superiority and effectiveness of our method, we use various evaluation criterions to evaluate the quantify of the images in Fig. 7, including local image contrast (IC)^24^, EME^25^, standard deviation^30^ σ and blind-reference-less image spatial quality evaluator (BRISQUE)^31^. Higher value of EME, IC and σ indicates higher image quality, while lower value of BRISQUE indicate higher image quality. The quantitative comparison of different recovered images is listed in Table 1. It can be seen from Table 1 that our method has the highest values of EME, IC and σ, while the lowest value of BRISQUE, which means that the performance of our method is better than others.

**Table 1:** Quantitative comparison of recovered images for the images in Fig. 7.

	BRISQUE	EME	IC	σ
Intensity image	42.10	0.26	0.06	0.06
Our	**11.54**	**2.10**	**0.42**	**0.46**
Schechner	16.29	1.21	0.22	0.30
HSI	29.80	1.57	0.25	0.28
HSS	12.27	1.66	0.36	0.29
He	31.37	1.20	0.27	0.18
CLAHE	40.92	0.98	0.17	0.10
Kim	31.08	1.62	0.36	0.34

**Figure 8:**
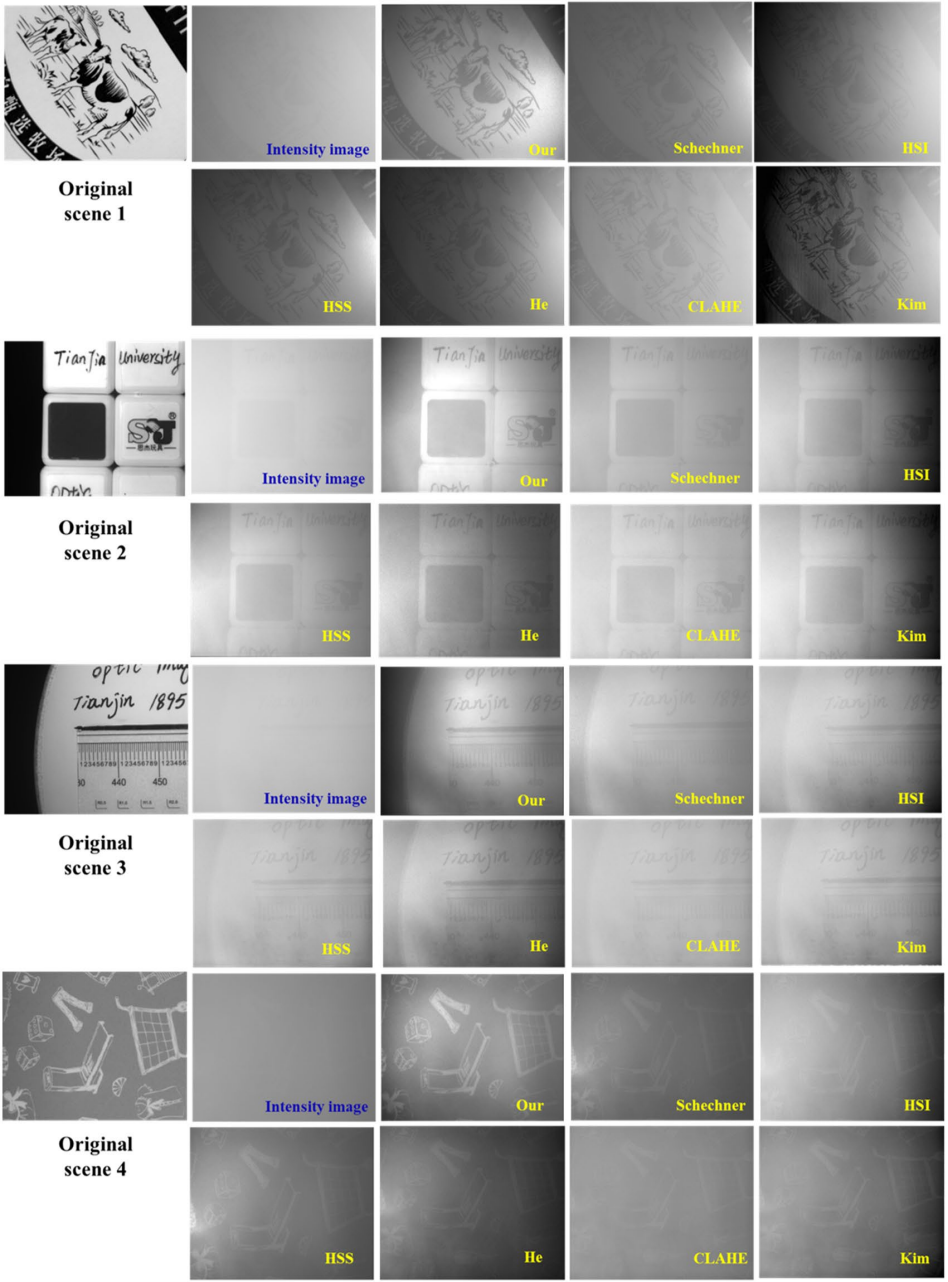
Comparison of the recovered scene by different methods (our method, Schechner’s method in ref.^17^, HIS (Histogram stretching for original intensity image); HSS (Histogram stretching after Schechner’s method), He’s method in ref.^14^, CLAHE in ref.^13^, Kim’s method in ref.^16^).

To demonstrate the universality of our method, we also perform the experiments of underwater image recovery for other four scenes with different densities of turbid medium. The results are shown in Fig. 8. It is shown that the recovered images by our method clearly reveal the scenes and the performance is better than other methods, which further demonstrate the effectiveness and superiority of the proposed method.

## Conclusion

In conclusion, we propose an improved method for underwater image recovery combining histogram stretching and polarimetric recovery in a proper way, which can significantly enhance the quality of underwater imaging. In the proposed method, the intensity histograms of the two orthogonal polarization images are stretched while maintaining the polarization relation, that is, the degree of polarization of the scene unchanged. Based on the two processed orthogonal polarization images, whose details are more distinguishable, a higher quality recovered image is obtained by the traditional polarimetric recovery method. The recovery results of real-world experiments demonstrate that it is feasible to enhance the quality of the image by our method. More importantly, the experimental results also demonstrate that the proposed method is quite effective in the condition of dense turbid medium.

The method proposed in this paper verifies the feasibility and effectiveness of combining computer vision method and polarimetric method for image recovery in turbid medium. Although histogram stretching method and Schechner’s polarimetric recovery method involved in this work are both old methods, the core idea of this combination has many perspectives. For example, future works can be performed to combine the more advanced computer vision methods (such as dark channel prior^14^ or deep learning^32^ etc.) with the more advanced polarimetric recovery methods (such as the polarization difference imaging^33^ or the multispectral polarization imaging^34^ etc.) to achieve the even better performance of the image recovery in turbid medium.
